# Analysis of Antioxidant Activity and Texture Profile of Tender-Young and King Coconut (*Cocos nucifera*) Mesocarps under Different Treatments and the Possibility to Develop a Food Product

**DOI:** 10.1155/2019/7470696

**Published:** 2019-02-25

**Authors:** S. Kalina, S. B. Navaratne

**Affiliations:** Department of Food science and Technology, University of Sri Jayewardenepura, Nugegoda, Colombo, Sri Lanka

## Abstract

The present study is aimed at analyzing the antioxidant activity using the free radical 2,2-diphenyl-1-picrylhydrazyl (DPPH) method and the texture using the Brookfield texture analyzer for blanched-solar dried, blanched-cooled with dehumidification, unblanched-solar dried, and unblanched-cooled with dehumidification mesocarps of two varieties of tender coconut, such as King coconut and Young coconut. Under the different treatments, there was a significant difference in the antioxidant activity and texture parameters such as “hardness” and “chewiness” at 5% level of significance. The highest (421.8 ± 12.33 mg/L) and the lowest (856.67±6.72 mg/L) antioxidant activities were recorded for blanched-solar dried mesocarp of King coconut and unblanched-cooled with dehumidification mesocarp of Young coconut, respectively. The texture analysis shows that there is a significant difference in the “hardness” and “chewiness” under different treatments at 5% level of significance. Among the treated mesocarps highest “hardness” value (6619.7±147.1) and “chewiness” value (1079.3±54.90) were recorded for King coconut blanched-solar dried mesocarp and lowest “hardness” value (595.67±36.88) and “chewiness” value (12.634±0.836) were recorded for Young coconut blanched-cooled with dehumidification mesocarp. Since the blanched-solar dried mesocarp of King Coconut has highest antioxidant activity and lowest “chewiness”, it is more suitable to develop a food product.

## 1. Introduction

There is a considerable interest in the consumption of particular foods to prevent the onset of diseases. Evidence suggests that diets rich in phenolic compounds can significantly improve and enhance the human health because of the effects of phenolic antioxidants [1]. Among them mainly fruits play a major role.* Cocos nucifera (*L), which belongs to the family Arecaceae, is commonly called the “coconut tree” and is known to be the most naturally wide spread fruit on earth [2]. Coconut (*Cocos nucifera *L.) is grown in about 93 countries; the most important cultivation areas and export countries are the Philippines, Indonesia, India, Malaysia, Sri Lanka, Mexico, and some of the islands in Oceania. Currently, about 90% of global supply comes from Asia where it is a prominent source of income for many countries [3].

Several studies have been carried out to identify the active compounds in coconut and their pharmacological and biological effects. Various extracts, fractions, and isolated compounds from different parts of the coconut fruit were analyzed, for showing different activities, such as antihypertensive; analgesia; vasodilation; protection against ulcers; protection of kidney, heart, and liver functions; and anti-inflammatory, antioxidant, antiosteoporosis, antidiabetes, antineoplastic, bactericidal, antimalarial, antihelminthic, antifungal, and antiviral activities [4].

There are two basic types of coconut based on the usage. One type is mainly used for obtaining coconut water at tender stage which is commonly called as King coconut and when maturing it shows poor nut development. The other type is the normal coconut; when maturing it will consist of well-developed nut which is used for producing desiccated coconut, coconut scrapings, coconut powder, coconut oil, etc. Sometimes the normal coconut in immature stage can be used for obtaining coconut water. This immature stage of normal coconut is known as Young coconut. The King coconut has the exterior skin colour of light orange and that of Young coconut is green. The outer shell of the nut is more thick and hard in normal coconut in mature stage. Normally coconut in immature stage is more nutritious and is one of the most distinct export fruits from the countries in Southeast Asia [5, 6].

There are various kinds of treatments applied to fruits to retain the nutritional quality and for preservation. Among them, Dried fruits are nutritionally equivalent to fresh fruits in smaller serving sizes. They have unique combination of taste/aroma, essential nutrients, fibre, and phytochemicals. More research should be performed to analyze the complete profiles of phytochemicals in fruits under drying process in relation to their antioxidant activities [7]. This study aims to analyze the antioxidant activity of tender coconut mesocarp under different treatments and take a step towards the development of a food product from the mesocarp.

## 2. Materials and Methods

### 2.1. Sample Preparation

The tender coconut fruits of the two varieties of coconut, Young coconut and King coconut, were taken and each of the coconut fruit is halved using a sharp curved knife. Then the mesocarp of the tender fruit was cut into small pieces using a sharp stainless steel knife. Then 4 treatments were given to the mesocarp pieces of the two varieties of coconut. These mesocarp pieces were used for the subsequent analysis of the study.

The four treatments are unblanched-solar dried where mesocarp pieces were dried in a tunnel solar drier at 70-75°C for 2 days, blanched-solar dried where mesocarp pieces were blanched with 0.5% citric acid at 95°C for 6 minutes and then dried in a tunnel solar drier at 70-75°C for 2 days, unblanched-cooled with dehumidification where cut pieces of coconut mesocarp were dried in a refrigerant humidifier at 4°C for 2 days, and finally blanched-cooled with dehumidification where cut mesocarp pieces were blanched with 0.5% citric acid at 95°C for 6 minutes. Then these blanched coconut mesocarp pieces were dried in a refrigerant humidifier at 4°C for 2 days.

To increase the effectiveness of the results, the coconut mesocarp samples with different treatments were prepared in triplicate using three separate coconut fruits of each variety.

### 2.2. Sample Extraction

For sample extraction, the method described by Appaiah et al. [8] was followed with some modifications. First the treated mesocarp was ground using the mortar and pestle into small pieces. Thereafter, 10.0000g ± 0.0001g of ground mesocarp pieces were weighed using the analytical balance PA214. Then the weighed sample was filled into the thimble and covered with cotton wool on the top and placed in the soxhlet apparatus. 250ml of 95% ethanol was added the round bottom flask and the extraction was carried out for 3 hours at 78.3°C.The extracted solvent was evaporated under vacuum at the temperature of 40-45°C using a rotary evaporator, to get the gummy concentrate of reddish colour.

### 2.3. Analysis of the Properties of Coconut Mesocarp

#### 2.3.1. Analysis of Antioxidant Activity

*Sample Stock Solution. *For each type treated mesocarp, the sample stock solutions having concentration of 0.01g/ml (10mg/ml) were prepared in 100ml volumetric flask by dissolving 1.0000 ± 0.0001g of extracted red gummy concentrate with methanol.

Using each of the solution prepared a dilution series was made by mixing 1 part of the sample stock solution to 4 parts of absolute methanol. From this dilution six different concentrations of sample solutions were prepared, having concentration of 2000mg/L, 400mg/L, 80mg/L, 16.mg/L, 3.2mg/L, and 0.64mg/L respectively.

*DPPH (1,1-dipheny-picryl hydrazyl) Radical Scavenging Activity. *For the DPPH radical scavenging activity, the method of Brand-Williams et al. [9] was followed by some modifications.

*(a) Preparation of DPPH Stock Solution. *Initially 0.0039g ±0.0001g (3.9mg) of DPPH chemical was weighed and the stock DPPH solution having concentration of 10^−4^ M was prepared using absolute methanol as the solvent and the resultant solution to have an absorbance of 0.900 ± 0.02 units at 517nm wave length, in UV-Vis spectrophotometer (UV mini -1240 model).

*∗*After preparing the DPPH solution, it was kept in dark room for 30 minutes before measuring the absorbance.

*(b) Determination of Antioxidant Activity. *A blank sample was prepared by mixing 0.5ml of methanol and 2.5ml of absolute methanol. A control sample was prepared by mixing 2.5ml of 10^−4^ M DPPH solution and 0.5ml of absolute methanol. From each dilution series of the sample, 0.5ml was taken and mixed with 2.5ml 10^−4^M DPPH stock solution. For all these volume measurements micropipette was used.

These mixtures were kept in dark place at room temperature for 15 minutes; then the resulting solution was vortexed for 30 seconds and the absorbance was read at 517nm in UV-visible spectrophotometer. Thereafter the percentage inhibition of absorbance was calculated for each dilution using the following equation.

(1) $$\begin{array}{c} \text{\%}\;\;inhibition=\left[\;\frac{Acontrol-Asample}{Acontrol}\;\right]x100 \end{array}$$

where* A control* is absorbance value of the DPPH solution of the control sample and* A sample* is absorbance value of the DPPH solution of the treated coconut mesocarp extract sample.

The calculated percentage of inhibition was plotted as a function of concentration of the coconut mesocarp extract sample. Then the concentration of sample, which gives 50% inhibition activity, was estimated as the IC_50_ value from regression analysis, using the software MINITAB®17.

For each concentration of the dilution series, the measurement of absorbance at 517nm was triplicated. As the sample (coconut mesocarp) is triplicated and the measurement is also triplicated, there will be nine absorbance values, inhibition%, and IC_50_ values for each concentration of coconut mesocarp extract in each type of treated mesocarp.

*(c) Drawing the Standard Curve. *The gallic acid which is a good antioxidant was used as the positive reference to the IC_50_ values of the sample. For this, six different concentrations of gallic acid such as 1, 2, 3, 4, 5, and 6 mg/L were prepared using absolute methanol as the solvent. The absorbance of each concentration of gallic acid was determined in the same way as done for the mesocarp samples.

The percentage of inhibition was calculated for each concentration of gallic acid solution and the percentage inhibition values were plotted as a function of concentration of standard antioxidant (gallic acid). This will impart the standard curve. The concentration of gallic acid solution which gives 50% inhibition activity was estimated as the IC_50_ value for standard reference in the same way as for the mesocarp.

#### 2.3.2. Analysis of Texture Profile of Coconut Mesocarps

In the texture profile analysis, the method described by Thomson et al. [10], was followed by some modifications.

Texture profile analysis (TPA) was performed with a view to analyze the mesocarp of the two coconut varieties under different treatments. The optimal test conditions set for the instrument were probe size of 4mm (T44), pretest speed of 2.00mm/s, test speed of 1.00mm/s, and posttest speed of 3.00mm/s. The target test parameter is the 75% deformation. The load was 1000g (10N). The TPA test is a two-cycle test.

After setting all the parameters in the texture Pro Ct software of the Brook field CT3 texture analyzer, the base of the instrument was located. After that each coconut mesocarp slice, having approximately 7mm thickness, was placed on the sample table of the texture profile analyzer and the load needed to make a 75% deformation in the mesocarp slice was determined by running the test and drawing a graph on load versus time using the texture Pro Ct software. Then the parameters needed for the analysis were selected and the results were recorded. The values of parameter such as hardness cycle-1, hardness cycle-2, and chewiness were recorded and analyzed.

The samples were triplicated and for each replicate five measurements were taken. Thus, each treated mesocarp of each coconut variety had 15 readings.

### 2.4. Data Analysis

The collected data was finally analyzed by using MINITAB®17 software. For the parametric data analysis. One-way ANOVA was used at 95% confidence interval and for the pair wise comparison of the means Tukey's Analysis was used. This was done to test whether there is a significant difference of antioxidant activity and the texture parameters among the different treatments of each variety of coconut. For the graphical representation of the data Microsoft Office Excel 2010 was used.

## 3. Results and Discussion

### 3.1. Analysis of Antioxidant Activity of Tender King and Young Coconut Mesocarp under Different Treatments

The ability of the natural antioxidant to scavenge the DPPH free radical will be measured by the reduction in absorbance by UV-visible spectrum at the wavelength of 517nm, and the IC_50_ value is the concentration of antioxidant components in the sample that could show 50% inhibition activity of the DPPH free radicals and it is indicated in the unit mg/L. Hence if lower concentration of the sample is required for half (50%) maximum inhibitory action, the sample has higher antioxidant activity.

The mean IC_50_ values of the DPPH assay done for the tender King and Young coconut mesocarp under different combination of treatments are given with their standard deviation values in Table 1.

As shown in Table 1, there was a significant difference among the IC_50_ values obtained for tender King and Young coconut mesocarps under different combination of treatments.

Further IC_50_ values were higher in Young coconut compared to King coconut and IC_50_ in unblanched mesocarp were higher than in blanched mesocarp.

But when comparing with the reference gallic acid (4.3180 ± 0.0117 mg/L) the antioxidant activity of treated mesocarp was lower. The reason may be that the synthetic antioxidants possess higher activity than the natural antioxidants, since the natural ones show a greater reluctance in donating hydrogen atoms when preventing oxidation [11].

Blanching is a heat treating process. According to Morales and Babel [12], four possibilities are there for the increase in antioxidant of some vegetables after cooking. They are the liberation high quantities of antioxidant components due to thermal destruction of cell walls and other cellular compartments; the production of a stronger radical, scavenging antioxidants by thermal chemical reaction; and/or suppression of the activity of antioxidants by thermal inactivation of oxidative enzymes; and production of new nonnutrient antioxidants or the formation of new compounds like products with antioxidant activity from Maillard reaction.

Several research studies have proved that blanching improves palatability and the bioavailability of antioxidants in vegetables [13]. Further, blanching would bring a number of changes in physical characteristics and chemical composition of vegetables [14, 15]. Oboh [16] stated that blanching had considerable effect on the contents of ascorbic acid and total phenolics and antioxidant activity of green leafy vegetables. Recent studies showed that blanching involving heat treatment reduces the antioxidant capacity in foods [17, 18].

Blanching of vegetable does not necessarily cause the loss of antioxidant properties. In some vegetables, blanching might actually improves the availability of the naturally occurring antioxidant components besides improving the palatability of the vegetable. Therefore, moderate blanching time and proper handling of vegetable are important in order to preserve the antioxidant properties [19].

According to Yamaguchii et al. [20], high antioxidant activity in boiled vegetables may occur as a consequence of the inactivation of oxidative enzymes such as ascorbate oxidase. However according to the results obtained it supports the fact blanching preserves the antioxidants in King and Young coconut mesocarp. In coconut mesocarp, blanching can inactivate the polyphenol oxidase enzymes which oxidize the phenolic compounds available in coconut mesocarp.

When considering the blanched mesocarps, for both King and Young coconut, solar dried mesocarp had higher antioxidant capacity than the cooled with dehumidification mesocarp. Among the unblanched mesocarps also solar dried mesocarps had higher antioxidant activity than the cooled with dehumidification mesocarp.

When comparing the solar dried mesocarp and cooled with dehumidification mesocarp, according to Chipurura et al. [21], thermal treatment reduces the antioxidants activity. In this study solar drying and cooling with dehumidifying were used as drying processes which removes moisture and inactivate the enzymes that lead to oxidation of poly-phenolic compounds. Solar drying is a thermal treatment and the cooling with dehumidifying is a nonthermal treatment. But according to the results obtained, solar dried mesocarps had high antioxidant activity than that cooled with dehumidification mesocarp. This may be due to the fact that, in cooling with dehumidifying, the enzymes are only inactivated; they can be activated when treated product is brought to room temperature. But in solar dried products, the solar heat will denature the enzymes in the coconut mesocarp. So, they cannot function again.

There is also evidence that ultraviolet (UV) light induces the synthesis of phenolics, including resveratrol, in skins of postharvested grapes exposed to UV-C for 2 min and these grapes have been proposed as a nutraceutical food [22]. Another reason may be that cooling with dehumidifying in this study was done with the help of a refrigerant dehumidifier. This may not be effective and advanced dehumidifying technique may be required.

In a previous study, the content of chlorophyll, flavonoid, and polyphenol were incremented in hot water blanched-solar dried samples as compared to other drying treatment like cabinet drying. Moreover, hot water blanching in combination with solar drying had higher polyphenol content in bitter-gourd [23]. The current study also supports the fact that blanched-solar dried samples of tender coconut mesocarp have the highest antioxidant capacity when compared to that cooled with dehumidification samples.

According to Meyer et al. [24], antioxidants activity of vegetables extracts also depends on the type and polarity at the extracting solvent, the isolation procedures, and purity of active compounds, as well as the assay techniques and substrate used. This factor may also have affected the results in different findings.

### 3.2. Analysis of the Textural Properties of King Coconut Mesocarp under Different Treatments

Texture is a prominent quality attribute of plant-based foods. The plant cell wall is a main determinant of texture in fruit and vegetables; its characteristics influence the way in which plant tissues undergo mechanical deformation during mastication. Processes, such as cooking, and physiological events such as ripening can decrease the strength of cell adhesion in many vegetables and fruit through depolymerization of pectin polysaccharides [25].

The mean hardness values of cycle 1 and cycle 2 and the chewiness values of the texture profile analysis test done for tender mesocarps of King and Young coconut under different treatments are given with their standard deviation in Table 2.

When considering the hardness cycle 1, hardness cycle 2, and chewiness, the P value obtained from ANOVA was 0.000. This was less than 0.05. So there was a significant difference in hardness of both cycles and chewiness among King coconut mesocarp under different treatments such as unblanched-solar dried, blanched-solar dried, unblanched-cooled with dehumidifying, and blanched-cooled with dehumidifying. Similarly there was a significant difference in hardness of both cycles and chewiness among Young coconut mesocarp under different treatments.

The hardness and chewiness of both King and Young coconut mesocarp under different treatments increase in the ascending order of blanched-cooled with dehumidification mesocarp, unblanched-cooled with dehumidification mesocarp, unblanched-solar dried mesocarp, and blanched-solar dried mesocarp. But according to Tukey's pairwise comparison, the difference between blanched-cooled with dehumidification and unblanched-cooled with dehumidification mesocarp in hardness was not significant for King coconut, but significant for Young coconut at 95% confidence levels and in chewiness was not significant for both King and Young coconut.

The mean values in Table 2 suggest that blanched-solar dried mesocarp of King coconut had the highest hardness in both cycle 1 and cycle 2 (6619g and 5298g, respectively). Blanched and cooled with dehumidification mesocarp had the lowest hardness, in both cycles 1 and 2 (1234g and 727g, respectively). For Young coconut also, it was the same where blanched-solar dried mesocarp of Young coconut had the highest hardness in both cycle 1 and cycle 2 (5435.3g and 4084.3g, respectively) and the blanched cooled with dehumidification mesocarp had the lowest hardness in cycles 1 and 2 (595.67g and 457.7g, respectively).

For the chewiness also blanched-solar dried mesocarp of King coconut had the highest chewiness (1079.3mJ) and the blanched-cooled with dehumidification mesocarp had the lowest chewiness (20.23mJ). For Young coconut also it was the same where blanched-solar dried mesocarp of Young coconut had highest chewiness (760.8mJ) and the blanched-cooled with dehumidification mesocarp of Young coconut had the lowest chewiness (12.63mJ). This may be due to the reason that solar drying process removes more water from the mesocarp than cooling with dehumidifying. If more water is removed the chewiness will be higher.

In most of the treated conditions both hardness cycles 1 and 2 are higher for King coconut variety than Young coconut. Among all the samples, blanched-solar dried mesocarp of tender King coconut had the highest hardness of cycles 1 and 2, and the blanched-cooled with dehumidification Young coconut mesocarp had the lowest hardness of both cycles 1 and 2.

Among all the treated conditions of the two varieties of coconut, the King coconut blanched-solar dried mesocarp had the highest chewiness and the Young coconut blanched-cooled with dehumidification mesocarp had the lowest chewiness.

When comparing the solar dried samples, the blanched-solar dried mesocarps had higher hardness than the unblanchedsolar dried mesocarps in both Young and King coconut. Blanching is a partial cooking process. Destruction of cell membranes occurs during heat processing and the turgor pressure is lost. Also cooking causes destruction along the middle lamella of cell walls [26]. So the cells will be softened on cooking [27] and the hardness will be reduced. So the available water in the cells comes out. Therefore, when blanched products are solar dried, there will be effective drying process evaporating more water from the product making the product harder. Hence the unblanched-solar dried coconut mesocarp had less hardness than blanched-solar dried mesocarp.

The same thing was observed when comparing the cooled with dehumidification mesocarps; unblanched-cooled with dehumidification mesocarp had higher hardness than blanched-cooled with dehumidification mesocarp. When these products are cooled with dehumidification after blanching, the moisture content will be reduced, but not as in that of solar drying.

When comparing to the solar dried mesocarp and cooled with dehumidification mesocarp, solar dried mesocarp had high hardness. According to Powers and Drake [28], asparagus loses textural acceptability when it is exposed to sunlight or held in the shade. So sunlight hardens the texture by removing water efficiently but cooling with dehumidifying is also drying process, but the removal of water from the mesocarp may not be efficient as that of solar drying. That is why solar dried mesocarps had higher hardness in both varieties of King coconut and Young coconut.

When comparing the chewiness among the treatments, for both Young coconut and King coconut, blanched mesocarps had lower chewiness than the un-blanched mesocarps and the solar dried mesocarps had higher chewiness than that cooled with dehumidification mesocarps. This may be due to the reason when the fresh mesocarp is solar dried water is removed more efficiently than cooling with dehumidifying, increasing the chewiness and on blanching the tissues will be softened and the chewiness will be reduced. Even though the blanched mesocarps are cooled with dehumidification for the drying purpose, it will not remove all the water as in the solar drying process. So chewiness will not increase much higher.

## 4. Conclusion

Antioxidant activity was expressed as IC_50_value which is the corresponding concentration giving 50% radical scavenging activity. The antioxidant analysis states that there was a significant difference in the antioxidant activity of both King and Young coconut under different treatments at 5% level of significance. The antioxidant activity of blanched mesocarps of both varieties is higher than unblanched mesocarps and the antioxidant activity solar dried mesocarp was higher than cooling with dehumidification mesocarps. This implies that the particular assay had responded well to antioxidants. The highest antioxidant activity (lower IC_50_) was recorded for blanched-solar dried mesocarp of King coconut, as 421.80±12.33, and lowest antioxidant activity (highest IC_50_) value was recorded for unblanched cooled with dehumidification Young coconut as 856.67±6.72 mg/L. So, it concludes that antioxidant activity depends on the variety of coconut and type of treatment.

The result of texture analysis shows that there was a significant difference in the hardness and chewiness at 5% level of significance and the hardness and chewiness values of both King and Young coconut mesocarps increases in the order of blanched-cooled with dehumidification mesocarp, unblanched-cooled with dehumidification mesocarp, unblanched-solar dried mesocarp, and blanched-solar dried mesocarp, respectively. In the hardness and chewiness values of corresponding treatments, King coconut was higher than the Young coconut. This concludes that texture of coconut mesocarp depends on variety of coconut and type of treatment.

Finally based on the result of antioxidant analysis and texture profile analysis, it could be concluded that the King coconut solar dried mesocarp was most suitable to develop a food product as it has the highest antioxidant activity and lowest chewiness.

In the world wide, people are motivated to consume food rich in antioxidants. The analysis of present study suggests that there are potentials to develop products by providing proper treatments that enhance the antioxidant capacity.

## Figures and Tables

**Figure 1:**
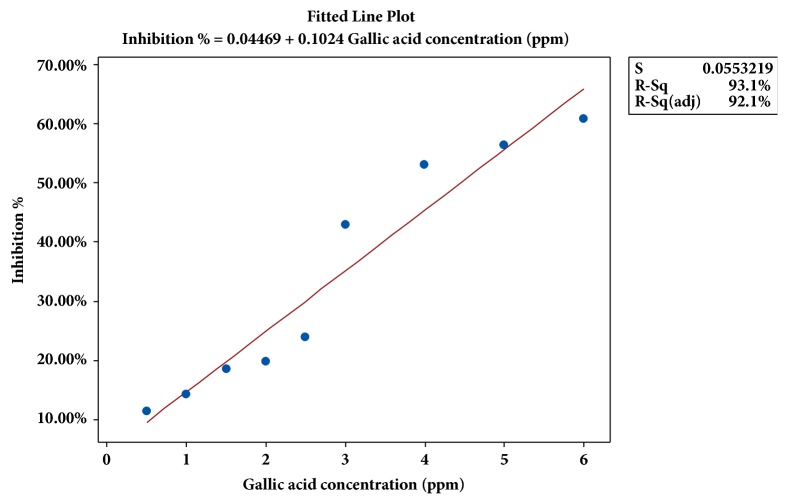
Standard curve for gallic acid.

**Table 1:** DPPH radical scavenging assay IC_50_ values in mg/L for tender King coconut mesocarp under different treatments.

Treatments	Un-blanched solar dried	Un-blanched-cooled with dehumidification	Blanched -solar dried	Blanched-cooled with dehumidification
King coconut	523.99 ± 9.38^c^	664.24 ± 9.62^d^	421.80 ± 12.33^b^	476.75 ± 4.90^a^

Young Coconut	711.12 ± 0.718^c^	856.67 ± 6.72^d^	511.94 ± 11.45^a^	644.05 ± 9.45^b^

*∗* Data presented as a mean values for triplicates with triplicate measurements in each replicate ± SD (n=9). a, b, c, and d letters in same row are significantly different at (P< 0.05) level.

**Table 2:** The hardness and chewiness values for King and Young coconut mesocarp under different treatments.

Treatments	Un-blanched solar dried mesocarp	Un-blanched cooled with dehumidification mesocarp	Blanched solar dried mesocarp	Blanched cooled with dehumidification mesocarp
*King Coconut*				
Hardness cycle-1(g)	5430.0±289.3^b^	1338.0±168.9^c^	6619.7±147.1^a^	1234.0±156.8^c^
Hardness cycle-2(g)	4165.7±135.5^b^	737.7±49.8^c^	5298.7±144.1^a^	727.3±49.2^c^
Chewiness (mJ)	308.84 ± 12.57^b^	45.96 ± 5.29^c^	1079.3 ± 54.90^a^	20.23 ± 4.00^c^
*Young Coconut*				
Hardness cycle-1(g)	3786.0±179.7^b^	814.7±147.0^c^	5435.3±170.0^a^	595.67±36.88^d^
Hardness cycle-2(g)	2502.00±26.17^b^	599.33±19.17^c^	4084.3±155.2^a^	457.7±39.5^d^
Chewiness (mJ)	140.88 ± 5.49^b^	23.875 ± 17.4^c^	760.8 ± 54.0^a^	12.634 ± 0.836^d^

*∗* Data presented as mean values for triplicates with five measurements in each replicate ± SD (n=15). a, b, and c letters in the same raw are significantly different at (P<0.05) level.

## Data Availability

The numerical data used to support the findings of this study are available from the corresponding author upon request.

## Appendix

See Figure 1.
